# Supplementary material for: From sequence to enzyme mechanism using multi-label machine learning
Source: BMC Bioinformatics. 2014 May 19;15:150. doi: 10.1186/1471-2105-15-150 (PMC4229970; doi:10.1186/1471-2105-15-150)
Supplement: Additional file 2 — Java code of ml2db. Additional file ml2db_code.tar.gz contains the Java source code to run the multi-label machine learning experiments and save the results to database. The code’s Javadoc is included. [file 1471-2105-15-150-S2.zip › additional file 2/ml2db/ml2db/doc/test/dataharness/LoadFirstValidArff.html]

LoadFirstValidArff


---


|  |  |  |
| --- | --- | --- |
| **Overview** \| **Package** \| **Class** \| **Use** \| **Tree** \| **Deprecated** \| **Index** \| **Help** | |  |
| **PREV CLASS**   **NEXT CLASS** | **FRAMES**    **NO FRAMES**     **All Classes** |  |
| SUMMARY: NESTED \| FIELD \| CONSTR \| METHOD | DETAIL: FIELD \| CONSTR \| METHOD |  |


---


## test.dataharness Class LoadFirstValidArff

```
java.lang.Object
  test.dataharness.LoadFirstValidArff
```

---

``` public class LoadFirstValidArff extends java.lang.Object ```

Class

**Version:**
:   7 Jul 2011

**Author:**
:   Luna De Ferrari luna.deferrari-at-ed.ac.uk

---

**Constructor Summary**

`LoadFirstValidArff()`


| **Method Summary** | |
| --- | --- |
| `static int` | `getFirstTestArffFromDb()` |
| `static mulan.data.MultiLabelInstances` | `getFirstTestArffInstancesFromDb()` |
| `static int` | `getFirstTrainArffFromDb()` |
| `static mulan.data.MultiLabelInstances` | `getFirstTrainArffInstancesFromDb()` |
| `static java.util.Vector<java.lang.String>` | `getFirstTrainPathFromDb()`             Get the arff path and xml path |
| `static java.lang.String` | `getTrainsetString()` |

**Methods inherited from class java.lang.Object**

`equals, getClass, hashCode, notify, notifyAll, toString, wait, wait, wait`

**Constructor Detail**

### LoadFirstValidArff

```
public LoadFirstValidArff()
```


**Method Detail**

### getFirstTestArffFromDb

```
public static int getFirstTestArffFromDb()
```

---


### getFirstTestArffInstancesFromDb

```
public static mulan.data.MultiLabelInstances getFirstTestArffInstancesFromDb()
```

---


### getFirstTrainArffFromDb

```
public static int getFirstTrainArffFromDb()
```

---


### getFirstTrainArffInstancesFromDb

```
public static mulan.data.MultiLabelInstances getFirstTrainArffInstancesFromDb()
```

---


### getFirstTrainPathFromDb

```
public static java.util.Vector<java.lang.String> getFirstTrainPathFromDb()
```

:   Get the arff path and xml path

    :   **Returns:**: the arff path (index 0) and xml path (index 1)

---


### getTrainsetString

```
public static java.lang.String getTrainsetString()
```


---


|  |  |  |
| --- | --- | --- |
| **Overview** \| **Package** \| **Class** \| **Use** \| **Tree** \| **Deprecated** \| **Index** \| **Help** | |  |
| **PREV CLASS**   **NEXT CLASS** | **FRAMES**    **NO FRAMES**     **All Classes** |  |
| SUMMARY: NESTED \| FIELD \| CONSTR \| METHOD | DETAIL: FIELD \| CONSTR \| METHOD |  |


---
